# Shared genomic features of HIV+ diffuse large B-cell lymphoma in two African cohorts

**DOI:** 10.1038/s41598-025-10529-6

**Published:** 2025-07-09

**Authors:** Sophia M. Roush, Mishalan Moodley, Jenny Coelho, Samantha Beck, Amon Chirwa, Edwards Kasonkanji, Marriam Mponda, Maurice Mulenga, Tamiwe Tomoka, Hanri van Zijl, Katherine Hodkinson, Arshad Ismail, Senzo Mtshali, Jonathan Featherston, Satish Gopal, Matthew S. Painschab, Jenifer Vaughan, Yuri Fedoriw

**Affiliations:** 1 Department of Pathology and Laboratory Medicine, School of Medicine, University of North Carolina (UNC), Brinkhous-Bullitt Building Rm 822 160 Medical Dr, Chapel Hill, NC 27514 USA; 2 Sequencing Core Facility, Division of the National Health Laboratory Service, National Institute for Communicable Diseases, Johannesburg, South Africa; 3 UNC Project Malawi, Lilongwe, Malawi; 4 Department of Molecular Medicine and Haematology, Faculty of Health Sciences, University of the Witwatersrand, Johannesburg, South Africa; 5 National Health Laboratory Services, Johannesburg, South Africa; 6 Department of Biochemistry and Microbiology, Faculty of Science, Engineering and Agriculture, University of Venda, Thohoyandou, South Africa; 7 Institute for Water and Wastewater Technology, Durban University of Technology, Durban, 4000 South Africa; 8 National Cancer Institute Center for Global Health, Rockville, MD USA; 9 UNC Lineberger Comprehensive Cancer Center, Chapel Hill, NC USA

**Keywords:** Cancer genetics, Predictive markers, B-cell lymphoma

## Abstract

The number of people with HIV (PWH) in Africa is rising due to population growth and antiretroviral therapy (ART) availability, with diffuse large B-cell lymphoma (DLBCL) a major cause of mortality. HIV and ART alter DLBCL tumor biology, but few studies of DLBCL include PWH or African patients, limiting translation of emerging treatment strategies. Here, we performed whole exome sequencing of 48 tumors (40 HIV-positive [HIV+]) with paired germline of DLBCL patients from Malawi and South Africa. HIV + DLBCL tumors had distinct mutations depending on ART exposure, and there were several recurrent deleterious variants, with *ANKRD11* mutations being prognostic. One tumor from each cohort had high tumor mutational burden and microsatellite-instability with *PMS2* and *ARID1A* mutation. These findings suggest shared genomic characteristics among HIV + DLBCL in Africa, offering opportunities for tailored biomarkers and therapeutic targets for this underserved population.

**Supplementary Information:**

The online version contains supplementary material available at 10.1038/s41598-025-10529-6.

## Introduction

Africa faces a rapidly growing cancer burden, driven by both population growth and the high prevalence of HIV^1^. By 2030, cancer incidence in Africa is expected to double, and by 2040, low- and middle-income countries are expected to account for 70% of global cancer deaths^2,3^. As cancer therapies increasingly depend on molecular and genomic characterization, disparities in translational research persist, with significant overrepresentation of patients of European ancestry^4,5^. These gaps are further exacerbated by the frequent exclusion of people with HIV (PWH) from translational and clinical studies of cancer, despite their growing proportion of cancer patients, particularly in Africa^6,7^.

In Eastern and Southern Africa, where more than half of the global HIV-positive (HIV+) population resides, the intersection of human immunodeficiency virus (HIV) and cancer has become a pressing public health issue^7^. The introduction of antiretroviral therapy (ART) has significantly improved life expectancy of PWH, yet cancer, particularly aggressive B-cell lymphomas like diffuse large B-cell lymphoma (DLBCL), has emerged as the leading cause of mortality among PWH^8–10^. Even when well-controlled on ART, PWH are over ten times as likely to develop DLBCL compared to people without HIV^11–14^. Before ART, survival rates for HIV + DLBCL patients were dismal, with overall survival (OS) rates below 20%, but current treatments combining full-dose chemotherapy and ART have significantly improved survival, making outcomes comparable to those in HIV-negative (HIV-) patients^11,15^.

Despite these clinical advances, genomic studies of HIV + DLBCL remain limited. While numerous studies of HIV- DLBCL have established genetic and transcriptomic subtypes, molecular characterization efforts in HIV + DLBCL have been constrained by sample numbers, absence of HIV- comparator groups, and restricted gene panel coverage^16,17^. Prior studies from our Malawi-based cohort provide evidence for impacts of HIV and ART on the transcriptomic profile, microenvironment, and T-cell receptor repertoire repertoires of DLBCL. Notably, established tumor biomarkers with prognostic significance in HIV- DLBCL, including cell-of-origin (COO), fail to correlate with HIV + DLBCL outcomes in Malawi, but whether this represents distinct tumor biology or reflects the impact of non-biologic factors remains unclear. This underscores the need for more inclusive research and population-specific biomarker validation^18–21^.

In addition to differences attributable to HIV and/or ART, studying multi-national cohorts may shed light on how ancestral, medical, and environmental factors influence molecular features and outcomes of DLBCL. Black DLBCL patients in the United States have inferior survival compared to white patients, and DLBCL patients of African ancestry are enriched for driver mutations in specific genes^22,23^. Yet, over 80% of tumor sequencing data originate from white individuals in high-income countries^4^.

To address the gaps in understanding of how HIV, ART exposure, and African ancestry influence DLBCL mutational profile, we performed whole exome sequencing on 48 DLBCL tumors and paired germline samples from cohorts in Malawi (MW) and South Africa (SA), including 40 HIV + cases. We aimed to characterize how HIV and ART shape the mutational profile of DLBCL—an extensively studied malignancy among HIV- individuals in high-income countries. Specifically, we compared clinical and histological features between HIV + and HIV- cohorts, assessed ART’s impact on the mutational landscape, and identified recurrent mutations and molecular characteristics in this historically understudied population.

## Methods

### Cohort characteristics

The MW cohort is composed of patients from the Kamuzu Central Hospital (KCH) Lymphoma Study (NCT02835911), which is based in Lilongwe, MW and began enrolling patients with newly diagnosed lymphoma in 2013^24,25^. Patients with histologically confirmed DLBCL receive CHOP (cyclophosphamide, doxorubicin, vincristine, and prednisolone) and a subset al.so received rituximab as part of a clinical trial (NCT02660710)^26^. Patients are followed for up to five years, with survival data censored on April 26th, 2024 for this study. The KCH Lymphoma Study was approved by the University of North Carolina IRB and Malawi National Health Sciences Research Committee, and all patients provide written informed consent. All studies were performed according to relevant guidelines and regulations. DLBCL patients enrolled from 2013 to 2021 with adequate pre-treatment tumor and paired whole blood samples for DNA extraction were included in this sequencing study.

The SA cohort is composed of patients diagnosed at or referred to Chris Hani Baragwanath Academic Hospital with a primary diagnosis of DLBCL between 2019 and 2022. The study was approved by the human research ethics committee of the University of Witwatersrand (M190709), and ethical clearance for next-generation sequencing was granted under reference number M220965. First-line therapy for patients with DLBCL in SA is CHOP, but several patients received rituximab following a suboptimal response (Table 1). Overall, 13 were treated with CHOP-based therapy, and three received rituximab as part of a second line regimen. Nine were either too ill to receive therapy or were treated with attenuated dose CHOP-based chemotherapy. Paired germline samples were sourced from uninvolved staging bone marrow aspirate slides.

**Table 1:** Clinical and tumor characteristics by HIV status and cohort.

	Malawi		South Africa	Total	
	HIV+ (*N* = 16)	HIV− (*N* = 8)	HIV+ (*N* = 24)	HIV+ (*N* = 40)	HIV− (*N* = 8)
Age (years)					
Median [Min, Max]	40.5 [17.0, 61.0]	46.0 [32.0, 57.0]	40.0 [19.0, 59.0]	40.5 [17.0, 61.0]	46.0 [32.0, 57.0]
Sex					
Female	5 (31.3%)	3 (37.5%)	12 (50.0%)	17 (42.5%)	3 (37.5%)
Male	11 (68.8%)	5 (62.5%)	12 (50.0%)	23 (57.5%)	5 (62.5%)
HIV/ART status					
HIV−	0 (0%)	8 (100%)	0 (0%)	0 (0%)	8 (100%)
HIV+/ART-exp	12 (75.0%)	0 (0%)	12 (50.0%)	24 (60.0%)	0 (0%)
HIV+/ART-naive	4 (25.0%)	0 (0%)	10 (41.7%)	14 (35.0%)	0 (0%)
CD4 count					
Median [Min, Max]	167 [32.0, 387]	NA	120 [23.0, 1120]	140 [23.0, 1120]	NA
HIV viral load					
Median [Min, Max]	0 [0, 37300]	NA	189 [20.0, 8320000]	61.0 [0, 8320000]	NA
B Symptoms					
No	8 (50.0%)	2 (25.0%)	4 (16.7%)	12 (30.0%)	2 (25.0%)
Yes	7 (43.8%)	6 (75.0%)	13 (54.2%)	20 (50.0%)	6 (75.0%)
Stage					
I–II	10 (62.5%)	2 (25.0%)	2 (8.3%)	12 (30.0%)	2 (25.0%)
III–IV	6 (37.5%)	6 (75.0%)	14 (58.3%)	20 (50.0%)	6 (75.0%)
ECOG score					
0–1	13 (81.3%)	3 (37.5%)	8 (33.3%)	21 (52.5%)	3 (37.5%)
2–4	3 (18.8%)	5 (62.5%)	9 (37.5%)	12 (30.0%)	5 (62.5%)
LDH					
Median [Min, Max]	453 [144, 1290]	427 [168, 845]	966 [271, 2500]	683 [144, 2500]	427 [168, 845]
COO (IHC)					
GC	13 (81.3%)	6 (75.0%)	11 (45.8%)	24 (60.0%)	6 (75.0%)
Non-GC	3 (18.8%)	2 (25.0%)	4 (16.7%)	7 (17.5%)	2 (25.0%)
Treatment					
Rituximab	2 (12.5%)	1 (12.5%)	3 (12.5%)	5 (12.5%)	1 (12.5%)
OS (months)					
Median [Min, Max]	26.2 [0.36, 60.0]	26.9 [0.66, 60.0]	2.38 [0.030, 44.0]	6.46 [0.030, 60.0]	26.9 [0.66, 60.0]

Clinical and immunohistochemical data of sequenced tumors stratified by cohort and HIV/ART status. CD4 T-cell count, HIV viral load and lactate dehydrogenase (LDH) laboratory values at diagnosis. ECOG = Eastern Cooperative Oncology Group score. Cell-of-origin: activated B cell (ABC), germinal center B cell (GCB) or unclassified subtype. Overall survival was censored at 60 months.

HIV + patients in both cohorts either continued or initiated ART. For the analyses in this study, HIV + patients with over six months of ART prior to study enrollment were considered ART-experienced (ART-exp). If ART duration was unknown or undocumented, HIV viral load < 200 was used to assign a patient as HIV+/ART-exp. Because only CD4 count was available, SA10 and SA15 were not assigned HIV/ART status and were not included in comparisons by HIV/ART status.

### Histologic diagnosis and immunohistochemistry

Primary histologic diagnoses were made per standard of care by pathologists and hematopathologists at Chris Hani Baragwanath Academic Hospital and the National Health Laboratory Service in SA, and as previously described at KCH^20,24,27,28^. COO was assigned for the MW cohort by immunohistochemistry (IHC) using CD10 and BCL6 (Leica Biosystems), and MUM1 (Dako)^28^. Based on IHC availability, COO was assigned for 15 samples from the SA cohort using CD10 (Roche), BCL6 (Cell Marque) and MUM1 (Dako).

### Whole exome sequencing

For the samples from MW, DNA was extracted from 40 pre-treatment formalin-fixed, paraffin-embedded (FFPE) DLBCL (GeneRead DNA FFPE Kit) and corresponding whole blood samples (QIAmp Blood Mini Kit) by Novogene Co (South Plainfield, NJ, USA). Target capture was performed using the Agilent SureSelect Human All Exon V6 r2 and the libraries were sequenced on the NovaSeq 6000 platform (Illumina, San Diego, CA). Three FFPE tumors had insufficient DNA quantity extracted (< 10 ng/ul), and seven FFPE tumors and seven blood samples failed Novogene’s library preparation or sequencing quality control. For the samples from SA, DNA was extracted from 24 FFPE DLBCL (Qiagen, AllPrep Kit) and corresponding uninvolved bone marrow aspirate (QIAmp Puregene Kit). Target capture was performed using the Agilent SureSelect Human All Exon V8 and the libraries were sequenced on the NextSeq 2000 platform (Illumina, San Diego, CA). One sample failed sequencing with < 10x average depth of coverage and was removed from the rest of the analysis (SA24).

### Somatic variant analysis

Tumor and normal FASTQs were processed using the jUNCtion pipeline (somatic-1.0.11, Agilent v6 profile, hg38, UNC Lineberger Bioinformatics Core) via the following steps: Quality control was performed using Picard (RRID: SCR_006525), MultipleMetrics (RRID: SCR_018929), and FastQC (RRID: SCR_014583). BAM files were realigned using coverageBed (RRID: SCR_006646). Variant calling was conducted with Strelka2 (RRID: SCR_005109), Cadabra (RRID: SCR_003277), and Mutect2 (RRID: SCR_000559), with results merged into a single VCF file using vcf2maf. QC output is available as recommended per Dreval et al.. in Supplemental Table 1^29^.

Only variants that passed all filters from the individual callers were included. MAFtools (RRID: SCR_024519) was used for further variant filtering and analyses, including oncoplot generation. Only genes mutated in both cohorts were included. Variants were included if they met the following criteria: tumor and germline read depth > 15, germline alternative count < 1, tumor alternative count > 5, and variant allele frequency (VAF) > 5%. Silent, intronic and common variants were excluded. A variant was considered to have an effect if likely deleterious or deleterious by SIFT (RRID: SCR_012813), likely damaging or damaging by PolyPhen (RRID: SCR_013189) and/or HIGH impact by VEP. Finally, TMB was calculated using MAFtools tmb function and reported as number of mutations after standard filtering.

### Epstein-Barr virus (EBV) status

EBV-encoded RNA in situ hybridization (EBER-ISH) was performed for MW samples to determine EBV status, with > 10% tumor staining considered positive. As EBER-ISH was not available for the tumors from SA, we performed Kraken taxonomic sequence classification (RRID: SCR_005484) on all tumors and used the MW tumors with both EBER-ISH and Kraken data as a benchmark.

### Tumor subtyping

LymphGen (https://llmpp.nih.gov/lymphgen/index.php) was applied to classify the tumors according to their genetic characteristics: BN2, EZB, MCD, N1, and ST2^17^. BCL2 and BCL6 translocation statuses were not available, and sequencing depth did not allow for copy number variant consideration (required for A53 subtype assignment).

### Microsatellite instability prediction and mismatch repair IHC

Microsatellite instability (MSI) was predicted using Microsatellite Analysis for Normal-Tumor InStability (MANTIS). MANTIS RepeatFinder was used for bed file generation (hg38) and bedtools intersect was used to filter for targets (Agilent v6 for MW samples, Agilent v8 for SA samples). MANTIS was run for all 48 paired tumor/germline samples (min read quality 20, min locus quality 25.0, min locus coverage 30, min repeat reads 1) and Step-Wise Difference above 0.4 was considered microsatellite instability-high^30^.

### Statistical tests

Wilcoxon rank-sum test was used to compare differences by cohort and HIV/ART status for continuous variables. Chi-square test was used to examine associations between categorical variables. Cox regression was used to calculate hazard ratio (HR) and p-value. Fisher’s exact test with false discovery rate (FDR) correction was used to determine differentially mutated genes. All statistical analyses were performed using R version 4.4.1.

## Results

### Comparison of clinical characteristics

**Fig. 1:**
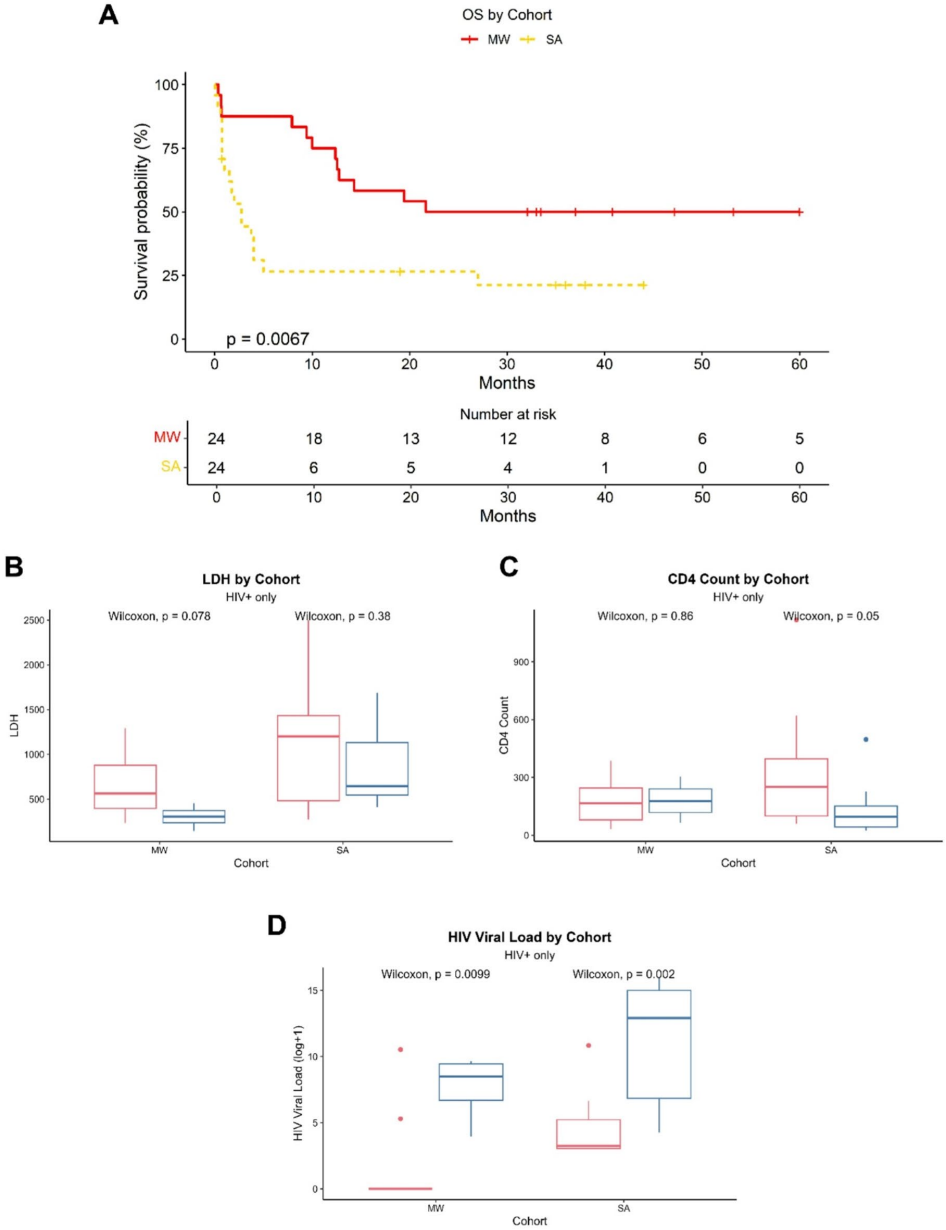
Clinical characteristics by HIV/ART status and cohort. (**A**) Overall survival (OS) by cohort. (**B**) Lactate Dehydrogenase (LDH) by HIV/ART status and cohort. (**C**) CD4 Count by HIV/ART status and cohort. (**D**) HIV viral load by HIV/ART status and cohort. (**B**–**D**) Comparisons by Wilcoxon rank-sum test, *n* = 40, HIV+ only. *MW* Malawi, *SA* South Africa.

Patients in the MW cohort had superior OS compared to those in the SA cohort (HR = 0.37, *p* = 0.008, Cox regression) (Fig. 1A). Further stratification by ART status demonstrated improved survival of HIV+/ART-naïve compared to HIV- patients in the MW cohort (HR = 0.58, *p* = 0.043, Cox regression), while ART status was not predictive of survival in the SA cohort (**Supplemental Fig. 1**). The proportion of patients with stage III or IV disease was higher in the SA cohort (*p* = 0.036, Chi square), although male/female ratios and the presence/absence of B symptoms were similar between the cohorts (Table 1). Lactate dehydrogenase (LDH) levels and European Cooperative Oncology Group (ECOG) performance status were higher in SA patients compared to MW patients (LDH: median 966 vs. 453, *p* = 0.001; ECOG: median 2 vs. 1, *p* = 0.028), even when considering only HIV + patients (LDH: median 982 vs. 453, *p* = 0.005; ECOG: median 2 vs. 1, *p* = 0.007) (**Supplemental Fig. 2**). In the MW cohort, there was a trend toward higher LDH levels in HIV+/ART-exp patients compared to HIV+/ART-naïve (*p* = 0.08, Fig. 1B). Meanwhile, in the SA cohort, HIV+/ART-naive patients had higher ECOG scores compared to HIV+/ART-exp (median 3 vs. 1, *p* = 0.024).

Among HIV + patients, CD4 counts were similar between cohorts (*p* = 0.93) but HIV viral load was higher in the SA cohort compared to MW cohort (median 188 vs. 0 copies/mL, *p* = 0.006) (**Supplemental Fig. 2A**,** B**). In the SA cohort, HIV+/ART-exp patients had higher CD4 counts compared to HIV+/ART-naïve (*p* = 0.05) and decreased HIV viral load (*p* = 0.002) (Fig. 1C, D). Meanwhile, in the MW cohort, only HIV viral load was significantly different by ART status (*p* = 0.01) (Fig. 1C, D). PWH in both cohorts received similar ART regimens, the most common being Tenofovir disoproxil (TDF), Lamivudine (3TC), Efavirenz (EFV) (7 MW, 9 SA) and the second most common being TDF/3TC/Dolutegravir (DTG) (3 MW, 5 SA). Three patients from the MW cohort received protease inhibitors (**Supplemental Fig. 2**).

### Commonly mutated genes in both cohorts

**Fig. 2:**
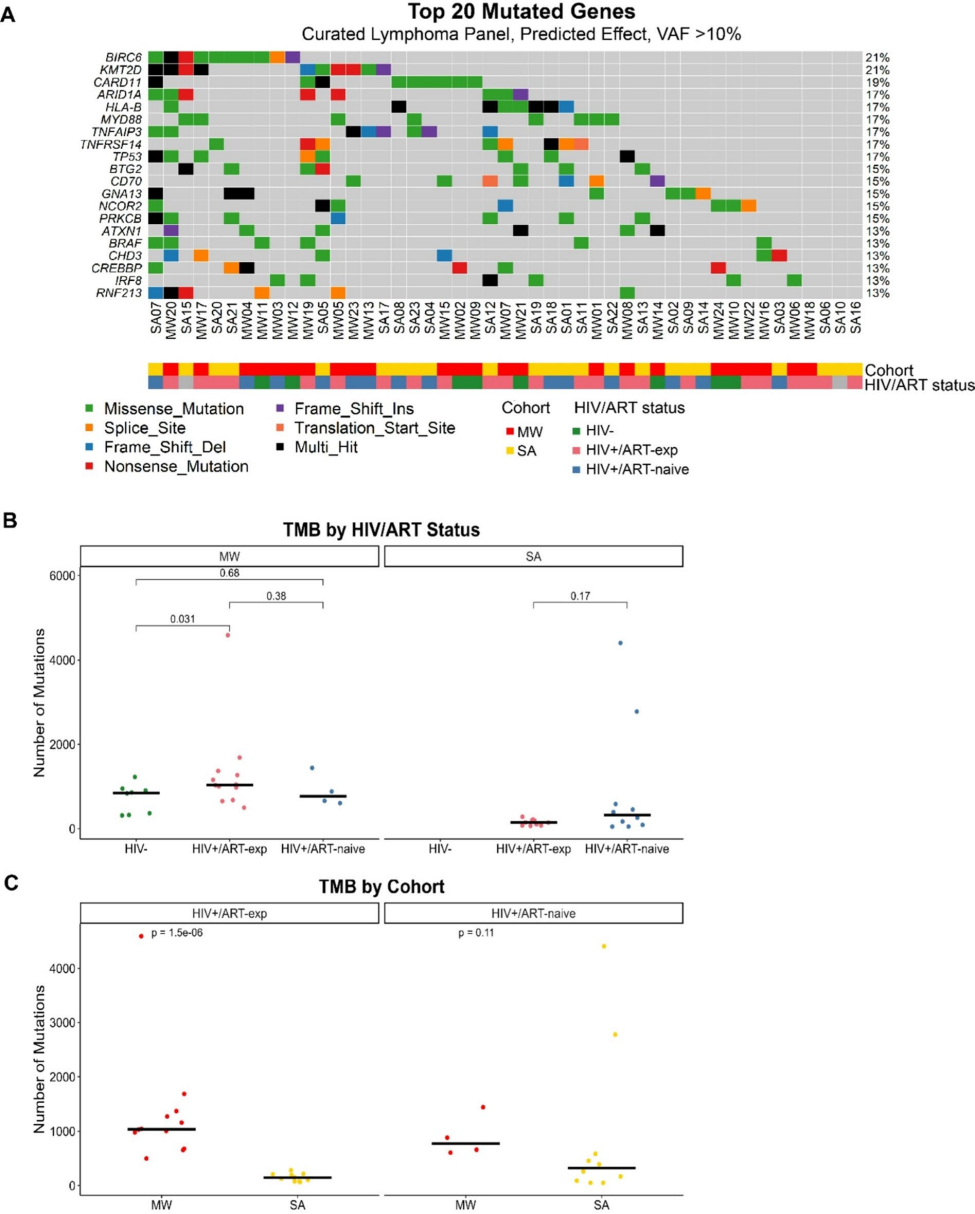
Recurrently mutated genes. (**A**) Oncoplot of the top 20 mutated genes across the combined cohort. Considering only genes from curated lymphoma panel, only variants predicted to have an effect with VAF > 10%^32^. (**B**) TMB by HIV/ART status, stratified by cohort. Pairwise Wilcoxon rank-sum test. (**C**) TMB by cohort, stratified by HIV/ART status. HIV+ tumors only. Pairwise Wilcoxon rank-sum test. (**B**, **C**) Whole exome, VAF > 5%, *n* = 38 HIV+ tumors with known ART status.

The median number of variants per sample was 498, with *TTN*, a large gene known to be frequently mutated in tumor exome studies, being the most recurrently mutated (62% samples).^31^ First, we used a curated lymphoma gene panel to determine the frequency of known DLBCL mutations in our combined cohort. Considering only variants predicted to have an effect with VAF > 10%, *BIRC6*,* KMT2D*, and *CARD11* were the most frequently mutated, with mutations in *ARID1A*,* MYD88*, and *TP53* also being common (Fig. 2A).

Although not validated or specifically developed for HIV + DLBCL, we applied the LymphGen genomic classification tool to our samples: 4 BN2, 1 BN2/MCD, 3 EZB, 1 EZB/MCD/ST2, 1 EZB/N1, 2 EZB/ST2, 1 MCD, 3 N1, 6 ST2, and the remaining 25 samples (53%) were assigned “Other” (**Supplemental Fig. 3**). Of genes included in the LymphGen classifier, *EZH2* was mutated in four MW samples (1 HIV-, 2 HIV+/ART-exp, 1 HIV+/ART-naïve), but no SA samples, and *MYC* was mutated in three SA samples (1 HIV+/ART-exp, 2 HIV+/ART-naïve), but no MW samples.

### South African HIV+/ART-exp tumors have lower TMB

MW samples had higher tumor mutational burden (TMB) overall (6-fold, *p* < 0.001), though samples from SA exhibited the second and third highest TMB values. There was no difference in TMB between HIV+/ART-exp and HIV+/ART-naïve DLBCL (*p* = 0.94). Within the MW cohort, HIV+/ART-exp tumors had higher TMB compared to HIV- (*p* = 0.031) (Fig. 2B). Comparing the cohorts, SA HIV+/ART-exp had lower TMB than MW HIV+/ART-exp (*p* < 0.001) (Fig. 2C).

### Differential mutations by HIV/ART status

**Fig. 3:**
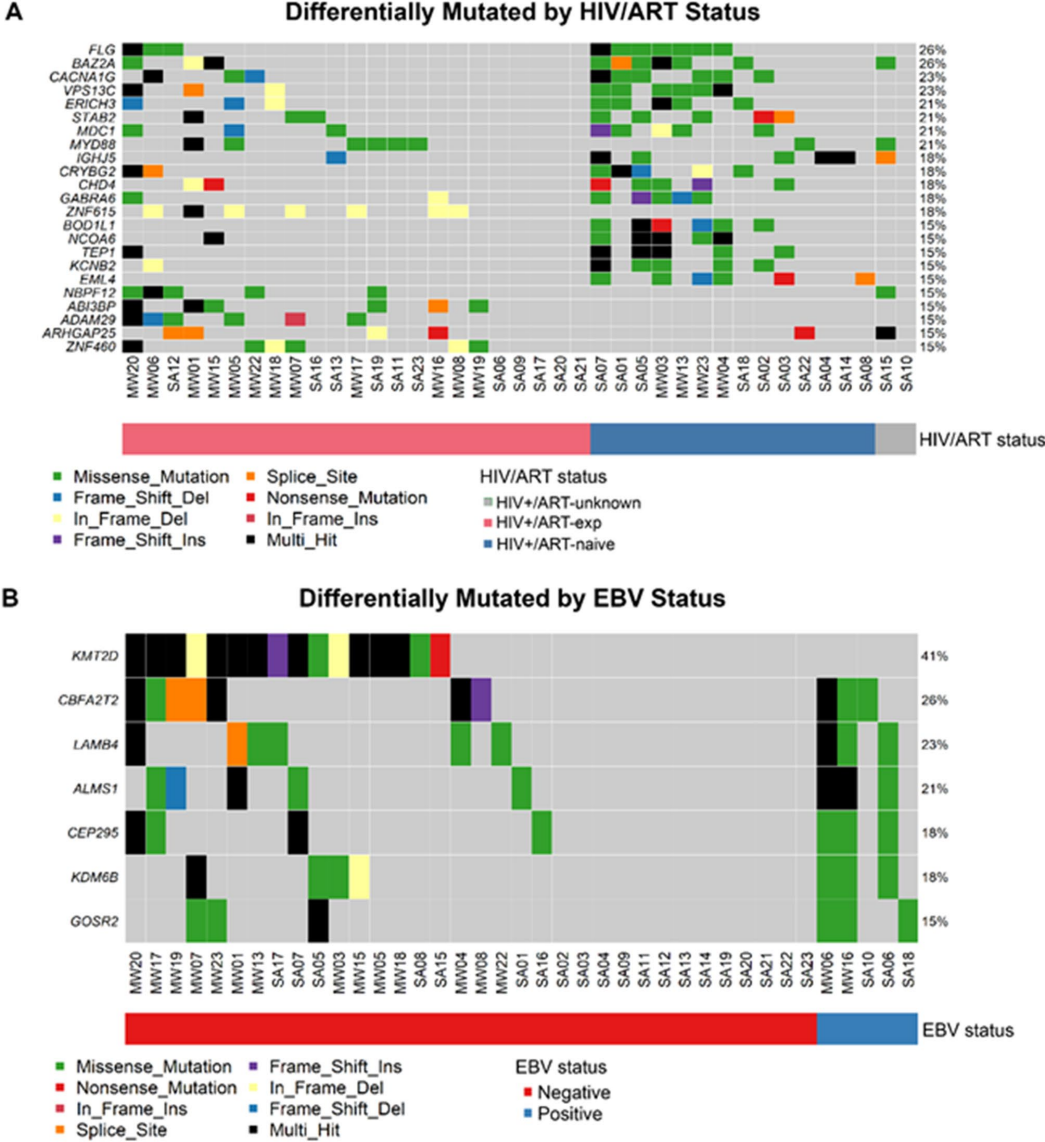
Differentially mutated genes by HIV/ART status and EBV status. (**A**) Differentially mutated genes by antiretroviral therapy (ART) status (FDR < 0.05, Fisher’s exact test). (**B**) Differential mutational profile by Epstein-Barr virus (EBV) status, as determined by Kraken (FDR < 0.1, Fisher’s exact test). (**A**, **B**) HIV+ tumors only, whole exome, VAF > 5%.

Gene mutations enriched in HIV+/ART-naïve compared to HIV+/ART-exp DLBCL included: *BOD1L1* and *EML4* (6/14 ART-naïve, 0/23 ART-exp, FDR = 0.001), *IGHJ5*, *NCOA6*, *TEP1*, *KCNB2*, (all 5/14 ART-naive, 1/23 ART-exp, FDR = 0.02), among others (Fig. 3A). Meanwhile, HIV+/ART-exp tumors were enriched for mutations in *DUSP2* (8/23 ART-exp, 0/14 ART-naïve, FDR = 0.02) and *PIM1* (7/23 ART-exp, 0/14 ART-naïve, FDR = 0.03) (Fig. 3A).

### EBV−/HIV + tumors enriched for KMT2D mutations

Computationally assigning EBV status using Kraken, six tumors had EBV read count > 200 and were considered EBV + for this study (**Supplemental Fig. 4**). EBV status was not associated with OS (*p* = 0.25), though EBV + patients in the MW cohort had comparatively poor survival (< 10 months) (**Supplemental Fig. 4**). EBV-/HIV + tumors were enriched for *KMT2D* mutations (0/5 EBV+, 16/34 EBV-, FDR = 0.07). Meanwhile, EBV+/HIV + tumors were enriched for several genes compared to EBV-/HIV + tumors including *GOSR2* (3/5 EBV+, 3/34 EBV-, FDR = 0.0192), *CEP295* (3/5 EBV+, 4/34 EBV-, FDR = 0.0321), and *KDM6B* (3/5 EBV+, 4/34 EBV-, FDR = 0.0321) (Fig. 3B). Of note, *STAT3* mutations were present exclusively in EBV-/HIV + tumors.

### Shared MYD88 variants among HIV+/ART-exp DLBCL across both cohorts

**Fig. 4:**
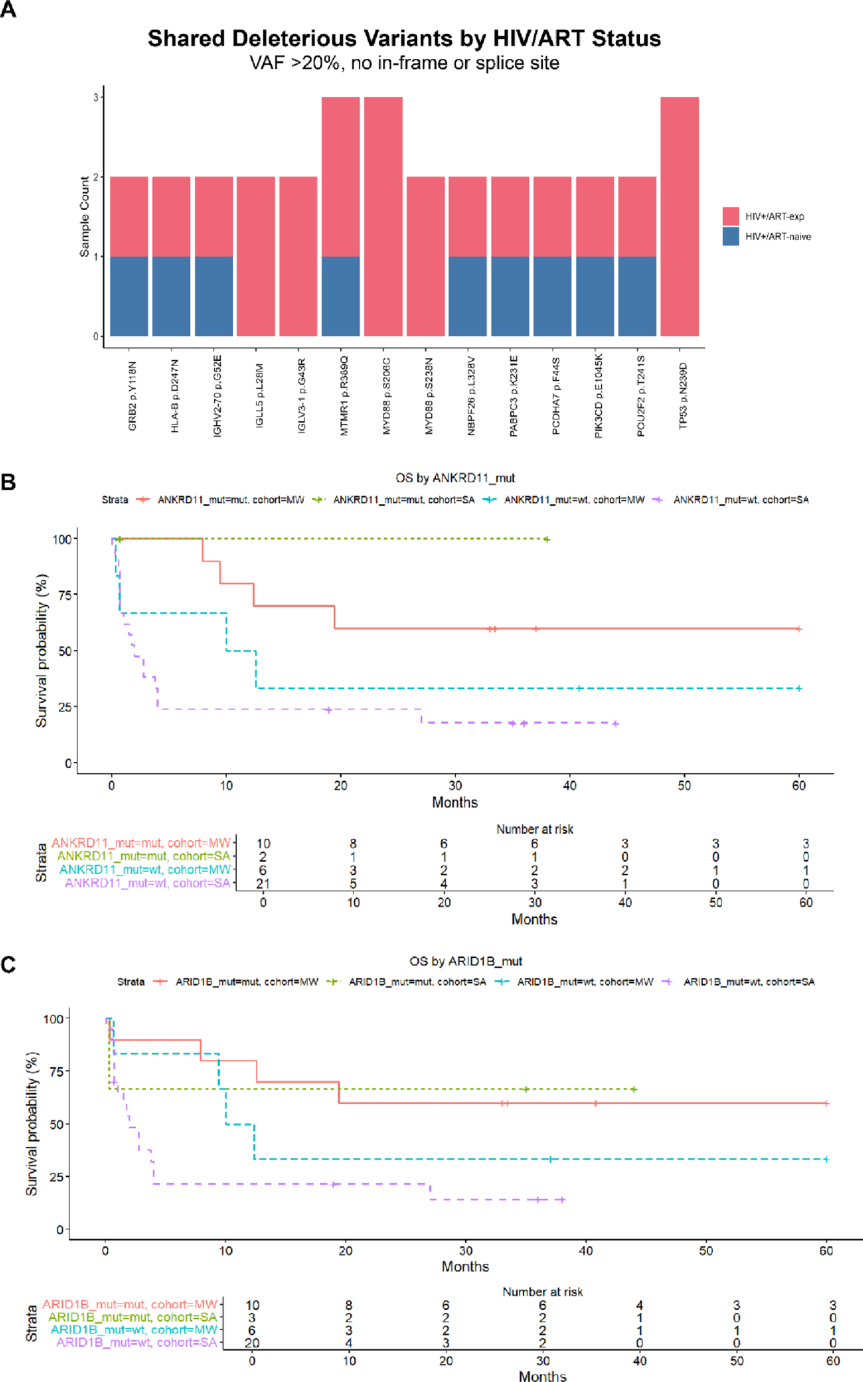
Shared and prognostic mutations among HIV + tumors. (**A**) Shared high variant allele frequency (VAF), predicted deleterious variants by antiretroviral therapy (ART) status. (**B**) Kaplan-Meier curve depicting overall survival (OS) by *ANKRD11* mutation status and cohort. (**C**) Kaplan-Meier curve depicting OS by *ARID1B* mutation status and cohort.

We identified shared pathogenic variants, defined as those predicted to be deleterious by SIFT or PolyPhen, with a variant allele frequency (VAF) exceeding 20% and excluding in-frame and splice site alterations, present in at least two tumor samples (Fig. 4A). Of these, five were present in at least one tumor from each cohort: *IGLL5* p.L28M, *MTMR1* p.R389Q, *MYD88* p.S206C, *PABPC3* p.K231E. *MYD88* p.S206C was found exclusively in HIV+/ART-exp tumors across the combined cohort, and *MYD88* p.S238N was found in two MW HIV+/ART-exp tumors.

### ANKRD11 mutations associated with improved survival in HIV + DLBCL

We investigated prognostic associations of genes mutated in at least five HIV + patients. Ten genes showed significant associations with OS (*p* < 0.05). Of these, five were mutated in two or more samples from each cohort: *MUC4*, *TTN*, *ANKRD11*, *ARID1B*, *RYR3*. Only *ANKRD11* (HR = 3.34, *p* = 0.054) and *ARID1B* (HR = 2.58, *p* = 0.086) mutations remained marginally prognostic after adjusting for cohort (Fig. 4B, C). Both *ANKRD11* (4.8-fold, *p* = 0.0087) and *ARID1B* (6.4-fold change, *p* < 0.001) mutation associated with higher TMB compared to non-mutated HIV + DLBCL samples.

### High mutational burden phenotype with microsatellite instability and PMS2 and ARID1A mutation

**Fig. 5:**
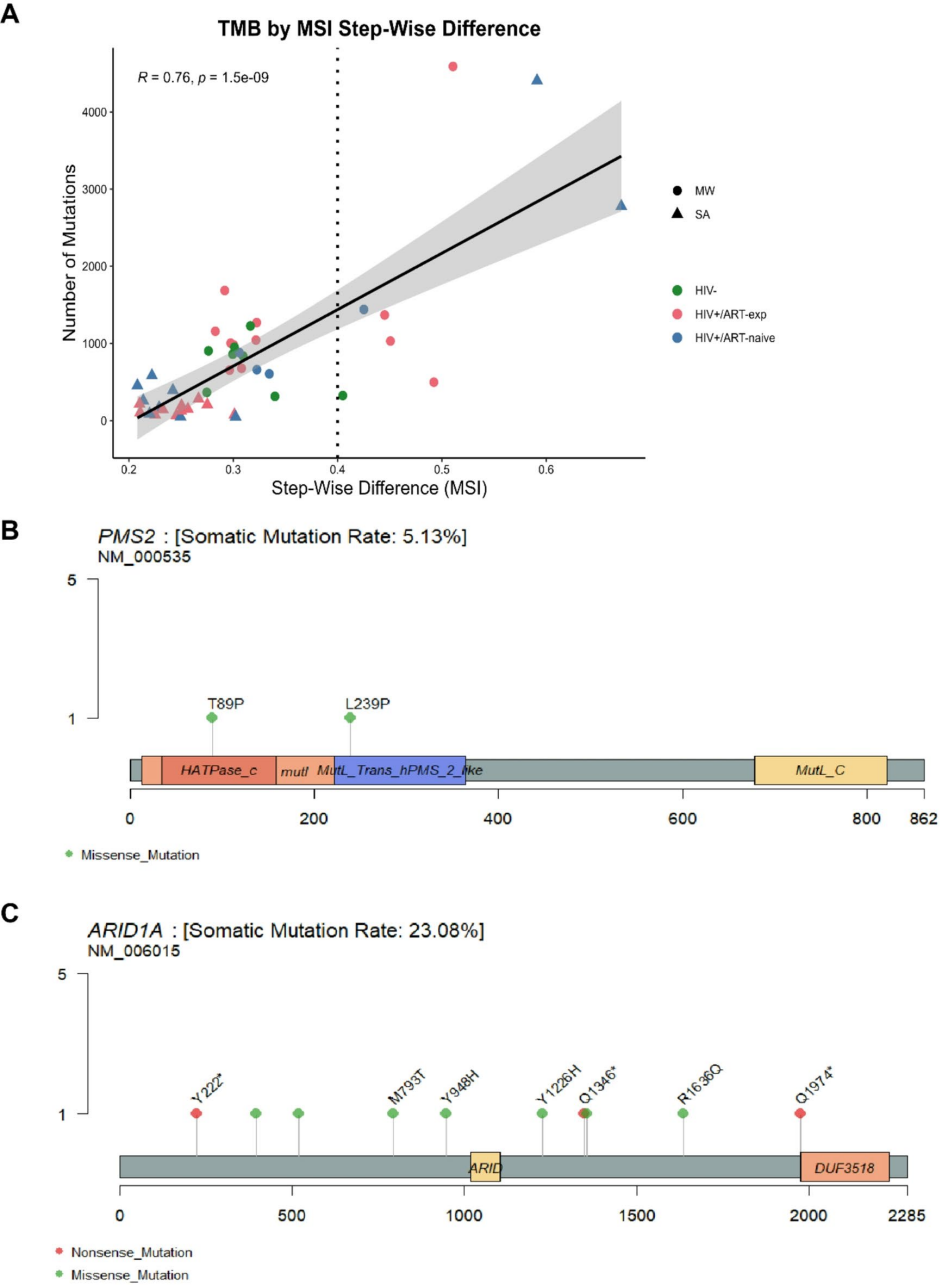
Evidence of microsatellite-instability among HIV + DLBCL. (**A**) Tumor mutational burden (TMB) by continuous microsatellite instability (MSI) metric using Pearson correlation. Dotted line indicates MSI-high (step-wise difference *≥* 0.4). Shape indicates cohort and color indicates HIV/ART status. *n* = 46, samples with known HIV/ART status. (**B**) Lollipop plot of *PMS2* mutations. C) Lollipop plot of *ARID1A* mutations. (**B**, **C**) All labelled variants occurred at a VAF > 10% and were predicted to have an effect.

Given several tumors exhibited over five times the median TMB, we assessed microsatellite instability (MSI) for all samples, and eight samples (6 MW, 2 SA) were predicted to be MSI-high, including the three tumors with the highest TMB (MW20, SA07 and SA05). Using a continuous metric, MSI correlated with TMB (*R* = 0.76, *p* < 0.001, Pearson correlation) (Fig. 5A). MSI-high tumors were enriched for *TTF2* mutations (6/8 MSI-high vs. 0/8 MSI-low, FDR < 0.001). We detected mutations in mismatch repair genes in 7/8 MSI-high samples. Interestingly, both MW20 and SA07 had *PMS2* (MW20: p.T89P VAF 63%, SA07: p.L239P VAF 44%) and *ARID1*A mutations (MW20: p.Y948H VAF 42% and p.Y518H VAF 9%, SA07: p.Y1226H VAF 36%) (Fig. 5B, C). Of note, both tumors harbored *ARID1A* mutations altering tyrosine to histidine and *PMS2* mutations changing to proline. MSI-high samples as well as additional samples with mutations in DNA mismatch repair genes or *ARID1A* are displayed in Table 2.

**Table 2:** Samples with mutations in DNA mismatch repair genes or *ARID1A*.

Sample	TMB	Variant (VAF)	HIV/ART status
MW20*	4591	*PMS2* p.T89P (62%)^#^ *ARID1A* p.Y948H (41%)^#^ *ARID1A* p.Y518H (9%)^#^	HIV+/ART-exp
SA07*	4407	*PMS2* p.L239P (44%)^#^ *ARID1A* p.Y1226H (36%)^#^	HIV+/ART-naive
SA05*	2778	*MLH1* p.A29G (63%)^#^ *MSH2* p.A636Hfs*49 (25%)^#^	HIV+/ART-naive
MW01	1686	*ARID1A* p.G397R (8%)^#^	HIV+/ART-exp
MW03*	1441	*MLH1* p.X196_splice (12%)^#^	HIV+/ART-naive
MW07*	1369	*ARID1A* p.R1636Q (21%)^#^ *MSH6* p.R298* (6%)^#^	HIV+/ART-exp
MW05	1271	*ARID1A* p.Q1346* (16%)^#^	HIV+/ART-exp
MW06*	1031	*MSH2* p.Q170E (44%)	HIV+/ART-exp
MW19	980	*ARID1A* p.Y222* (12%)^#^	HIV+/ART-exp
MW24	951	*MSH6* p.L743Ffs*13 (35%)^#^	HIV-
MW12	860	*MSH2* p.R96H (7%)^#^	HIV-
MW11	837	*ARID1A* p.T1359_Q1367del (5%)	HIV-
MW16	678	*MSH2* (12%)^#^	HIV+/ART-exp
MW04	660	*ARID1A* p.F1354S (7%)^#^	HIV+/ART-naive
MW21*	325	*MSH6* p.A587P (40%)^#^ *ARID1A* p.Q507Vfs*117 (24%)^#^	HIV-
SA12	283	*ARID1A* p.M793T (62%)^#^	HIV+/ART-exp
SA15	165	*ARID1A* p.Q1974* (35%)^#^	HIV+/ART-unknown

*MSI-high by MANTIS.

^#^Variant predicted to have an effect.

## Discussion

This study offers the first multinational somatic mutational profiling of HIV + DLBCL from Africa, advancing our understanding of a patient population historically underrepresented in genomic profiling studies. By integrating data from patient cohorts in MW and SA, we identified shared genomic variants and microsatellite instability in HIV + DLBCL, and further suggest that ART exposure may impact the mutational profile. These findings add to biological understanding of lymphomagenesis in the context of HIV and ART, and are essential for ensuring PWH benefit from emerging therapies minimize cytotoxicity and improve outcomes for patients with relapsed/refractory disease.

Clinical parameters differed significantly between the two cohorts, with SA patients showing poorer HIV control, elevated LDH, higher ECOG scores at diagnosis, and inferior survival. Although this study did not have an epidemiological focus, these observed disparities in clinical outcomes between the cohorts likely stem from variations in access to HIV care, healthcare infrastructure, and time to diagnosis and treatment. Previous studies have shown contrasting survival patterns: MW HIV+/ART-naïve patients demonstrated better survival compared to HIV+/ART-exp patients, while SA data showed the opposite pattern^19,33^. It is possible that the analyzed MW cohort may be enriched for HIV+/ART-naïve individuals with favorable biologic characteristics who survive long enough to access care. Additionally, exclusive presence of *EZH2* mutations in the MW cohort and *MYC* mutations in the SA cohort may provide further insight or support for these survival disparities and warrant further investigation^34^.

The differential mutation patterns between HIV+/ART-exp and HIV+/ART-naïve tumors supports the hypothesis that ART exposure shapes the mutational profile of HIV + DLBCL. HIV+/ART-exp tumors were enriched for *DUSP2* and *PIM1* mutations, among others. In contrast, HIV+/ART-naïve tumors displayed a distinct profile with mutations in *BOD1L1* and *EML4*, both DNA damage response genes. These findings support prior evidence that HIV infection promotes lymphomagenesis through increased DNA damage, highlighting a potential mechanistic link between HIV and lymphoma development^35^. Although EBV is commonly assumed to be implicated in many HIV + DLBCL cases, only 15% of HIV + tumors were EBV + in this study^36^. Among the HIV + tumors, EBV- tumors were enriched for *KMT2D* mutations, consistent with a prior study identifying histone-modifying gene mutations in EBV-/HIV + DLBCL.^32^ However, unlike previous reports, *STAT3* mutations in our cohort occurred exclusively in EBV- cases^32^.

Our analysis also identified several shared, high VAF deleterious variants across both cohorts, including *IGLL5* (p.L28M), *MYD88* (p.S206C), and *MTMR1* (p.R389Q) which only occurred in HIV+/ART-exp tumors. These shared variants suggest there are common mutational drivers in HIV + DLBCL in southern Africa, which may represent therapeutic targets or biomarkers for this population. Additionally, *ANKRD11* mutations were positively prognostic in our study, consistent with findings from a study of EBV + HIV- DLBCL^37^. Interestingly, while the latter study found *ANKRD11* mutations in 32% of EBV + HIV- DLBCL, none of the *ANKRD11* mutations in our cohorts were found in EBV + tumors, indicating dysregulation of a more general immune mechanism, rather than an EBV-specific one^37^.

Finally, the identification of two HIV + tumors across the two cohorts, both characterized by high TMB and MSI, with similar predicted pathogenic mutations in *ARID1A* and *PMS2 —*genes associated with DNA mismatch repair deficiency*—* is particularly intriguing^38,39^. MW20 was HIV+/ART-exp with 37 months of ART exposure prior to DLBCL diagnosis, and SA07 was HIV+/ART-naïve but defaulted on their ART regimen prior to diagnosis, indicating they might have had substantial exposure to ART. These findings are consistent with previous work linking DNA damage to chronic immune dysregulation as driver for lymphomagenesis, and define a subset of HIV + DLBCL patients that may benefit from targeted therapies^40–43^.

In conclusion, this study begins to fill the critical gap in our understanding of the molecular mechanisms driving HIV + DLBCL in Africa. Our findings highlight the importance of ART exposure in shaping the mutational landscape of HIV + DLBCL, with potential implications for developing tailored therapeutic strategies. Understanding the role of DNA repair defects in HIV + DLBCL could inform strategies for selecting patients who may benefit from therapies targeting DNA damage, such as PARP inhibitors or other DNA repair modulators. However, the relatively small number of cases, differences in health systems, and potential selection biases limit the generalizability of our results to all HIV + DLBCL patients across the continent. Broader studies including additional geographic regions and larger sample sizes will be essential to confirm these observations and assess their relevance to the wider population. As the burden of HIV in Africa remains high, these insights are crucial for ensuring that emerging therapies are accessible and effective for HIV + DLBCL patients in low-resource settings.

## Electronic supplementary material

Below is the link to the electronic supplementary material.

Supplementary Material 1

Supplementary Material 2

Supplementary Material 3

## Data Availability

Filtered variants are included as supplemental data, genomic data from the Malawi and South Africa cohorts are available in the BioProject repositories, PRJNA1203246 and PRJNA1281316, respectively. Code available by request from corresponding author.
