# Correction: Shared genomic features of HIV+ diffuse large B-cell lymphoma in two African cohorts

**DOI:** 10.1038/s41598-026-38395-w

**Published:** 2026-02-11

**Authors:** Sophia M. Roush, Mishalan Moodley, Jenny Coelho, Samantha Beck, Amon Chirwa, Edwards Kasonkanji, Marriam Mponda, Maurice Mulenga, Tamiwe Tomoka, Hanri van Zijl, Katherine Hodkinson, Arshad Ismail, Senzo Mtshali, Jonathan Featherston, Satish Gopal, Matthew S. Painschab, Jenifer Vaughan, Yuri Fedoriw

**Affiliations:** 1 Department of Pathology and Laboratory Medicine, School of Medicine, University of North Carolina (UNC), Brinkhous-Bullitt Building Rm 822 160 Medical Dr, Chapel Hill, NC 27514 USA; 2 Sequencing Core Facility, Division of the National Health Laboratory Service, National Institute for Communicable Diseases, Johannesburg, South Africa; 3 UNC Project Malawi, Lilongwe, Malawi; 4 Department of Molecular Medicine and Haematology, Faculty of Health Sciences, University of the Witwatersrand, Johannesburg, South Africa; 5 National Health Laboratory Services, Johannesburg, South Africa; 6 Department of Biochemistry and Microbiology, Faculty of Science, Engineering and Agriculture, University of Venda, Thohoyandou, South Africa; 7 Institute for Water and Wastewater Technology, Durban University of Technology, Durban, 4000 South Africa; 8 National Cancer Institute Center for Global Health, Rockville, MD USA; 9 UNC Lineberger Comprehensive Cancer Center, Chapel Hill, NC USA

Correction to: *Scientific Reports* 10.1038/s41598-025-10529-6, published online 09 July 2025

In the original version of the Article, Figure 4 was a duplication of Figure 3.

The original Article has been corrected.

