# Supplementary material for: Shared genomic features of HIV+ diffuse large B-cell lymphoma in two African cohorts
Source: Sci Rep. 2025 Jul 9;15:24599. doi: 10.1038/s41598-025-10529-6 (PMC12241603; doi:10.1038/s41598-025-10529-6)
Supplement: Supplementary file 2 — Supplementary Material 2 [file 41598_2025_10529_MOESM2_ESM.pdf]

Shared genomic features of HIV+ diffuse large B-cell lymphoma in two African cohorts

Running Title: Shared genomic features of HIV+ DLBCL in Africa

Sophia M. Roush[1*], Mishalan Moodley[2]*, Jenny Coelho[1], Samantha Beck[1], Amon Chirwa[3], Edwards Kasonkanji[3], Marriam Mponda[3], Maurice Mulenga[3], Tamiwe Tomoka[3], Hanri van Zijl[4,5], Katherine Hodkinson[4,5], Arshad Ismail[2,6,7], Senzo Mtshali[2], Jonathan Featherston[2], Satish Gopal[8], Matthew S. Painschab[9], Jenifer Vaughan[4,5]*, Yuri Fedoriw*[1,9]

[1]Department of Pathology and Laboratory Medicine, School of Medicine, University of North Carolina (UNC), Chapel Hill, NC, USA, [2]Sequencing Core Facility, National Institute for Communicable Diseases, Division of the National Health Laboratory Service, Johannesburg, South Africa, [3]UNC Project Malawi, Lilongwe, Malawi, [4]Department of Molecular Medicine and Haematology, Faculty of Health Sciences, University of the Witwatersrand, Johannesburg, South Africa, [5]National Health Laboratory Services, Johannesburg, South Africa, [6]Department of Biochemistry and Microbiology, Faculty of Science, Engineering and Agriculture, University of Venda, Thohoyandou, South Africa, [7]Institute for Water and Wastewater Technology, Durban University of Technology, Durban 4000, South Africa, [8]National Cancer Institute Center for Global Health, Rockville, MD, USA, [9]UNC Lineberger Comprehensive Cancer Center, Chapel Hill, NC, USA

* authors contributed equally

Corresponding author:
Yuri Fedoriw
Brinkhous-Bullitt Building Rm 822
160 Medical Dr, Chapel Hill, NC 27514

yuri.fedoriw@unchealth.unc.edu

Supplemental Figure 1. Overall survival by HIV/ART status and cohort.

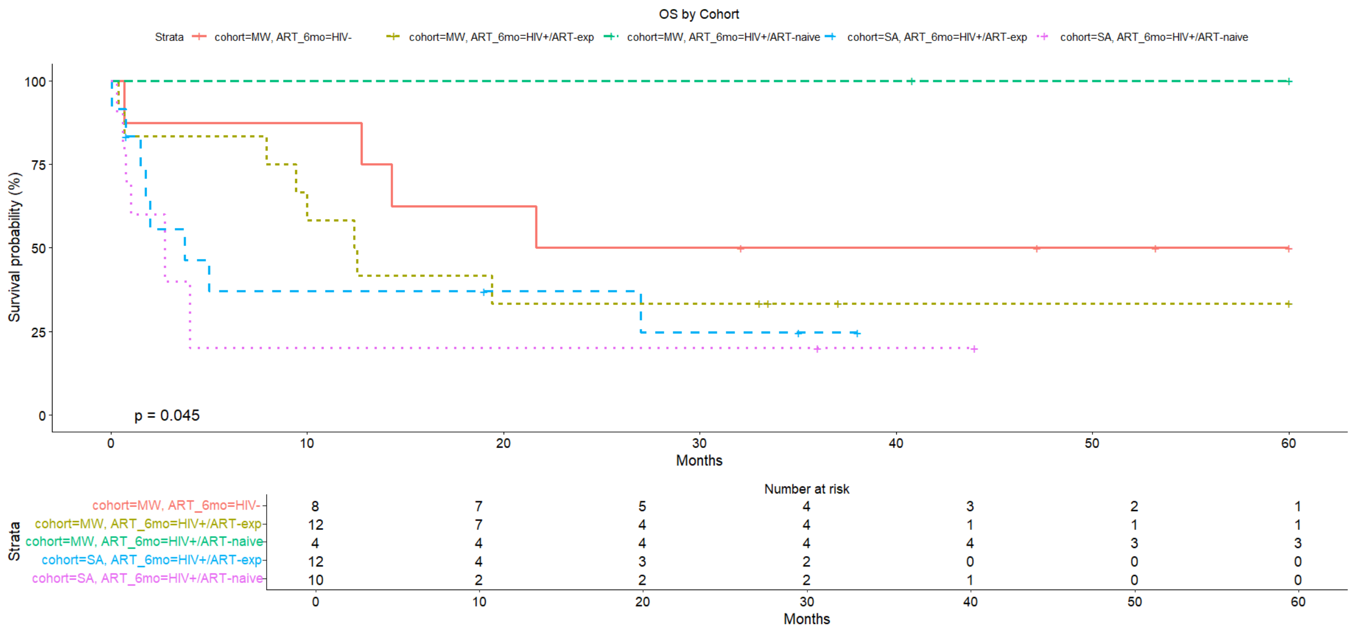

Kaplan-Meier curve of overall survival stratified by cohort and HIV/ART status.

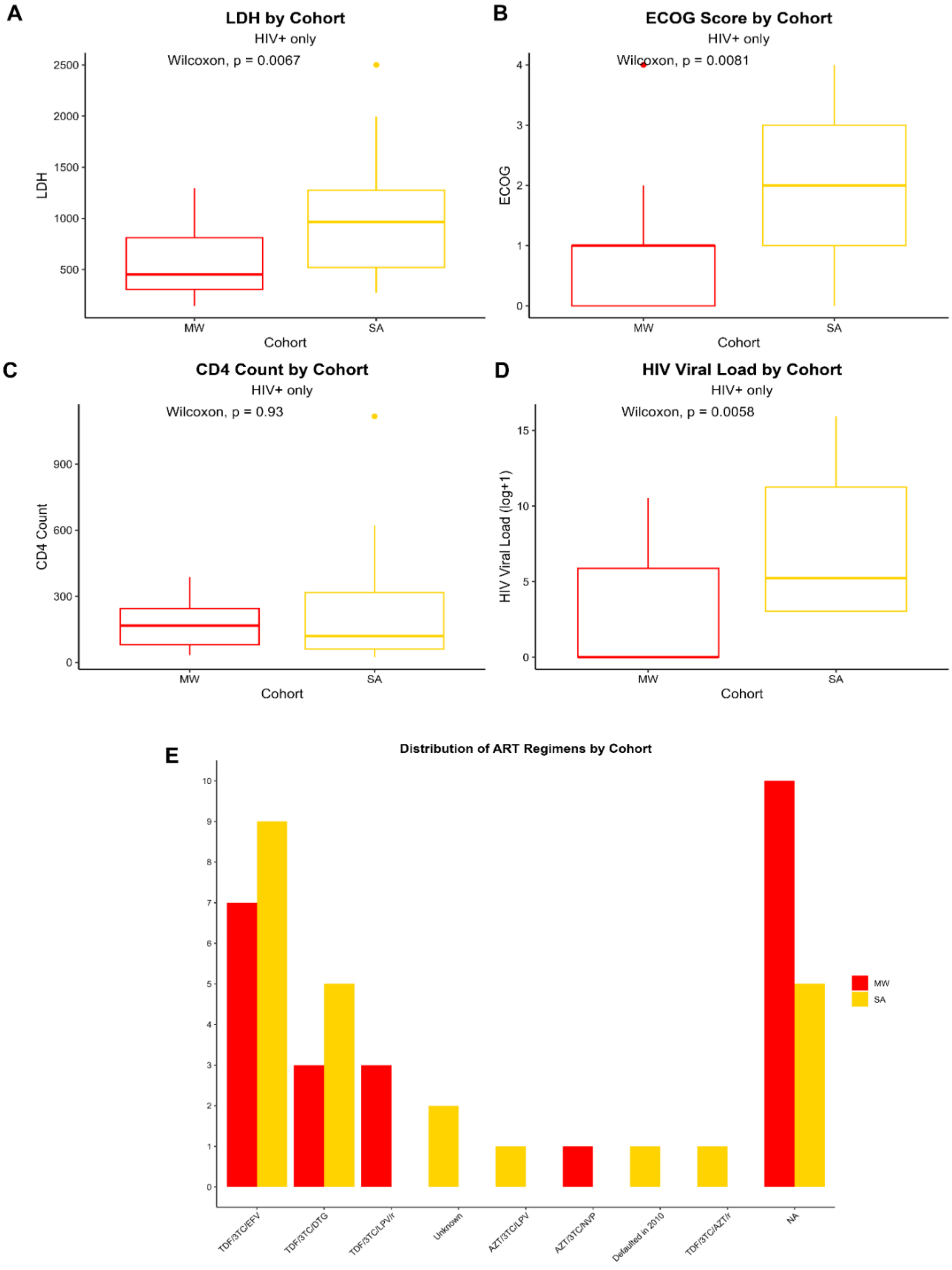

Supplemental Figure 2. Clinical characteristics at diagnosis by cohort. **A)** CD4 count by cohort. **B)** HIV viral load by cohort. **C)** Lactate Dehydrogenase (LDH) by cohort. **D)** European Cooperative Oncology Group (ECOG) score by cohort. **E)** ART regimen by cohort. **A-D**: Wilcoxon rank-sum test.

A

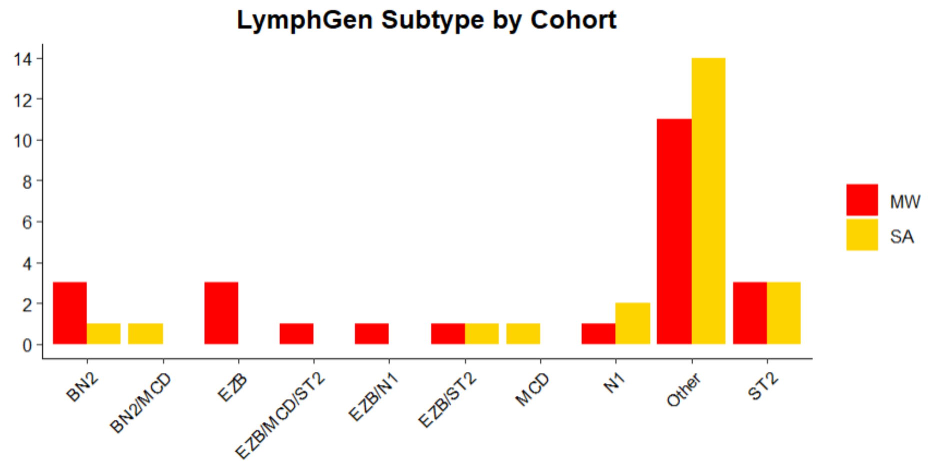

B

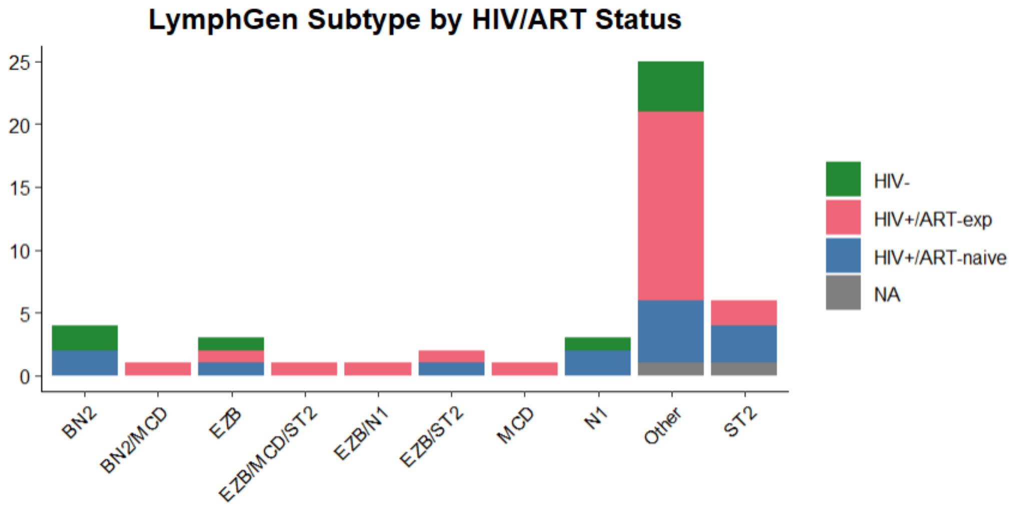

Supplemental Figure 3. LymphGen genetic subtyping. A) Subtype assignment by cohort. **B)** Subtype assignment by HIV/ART status.

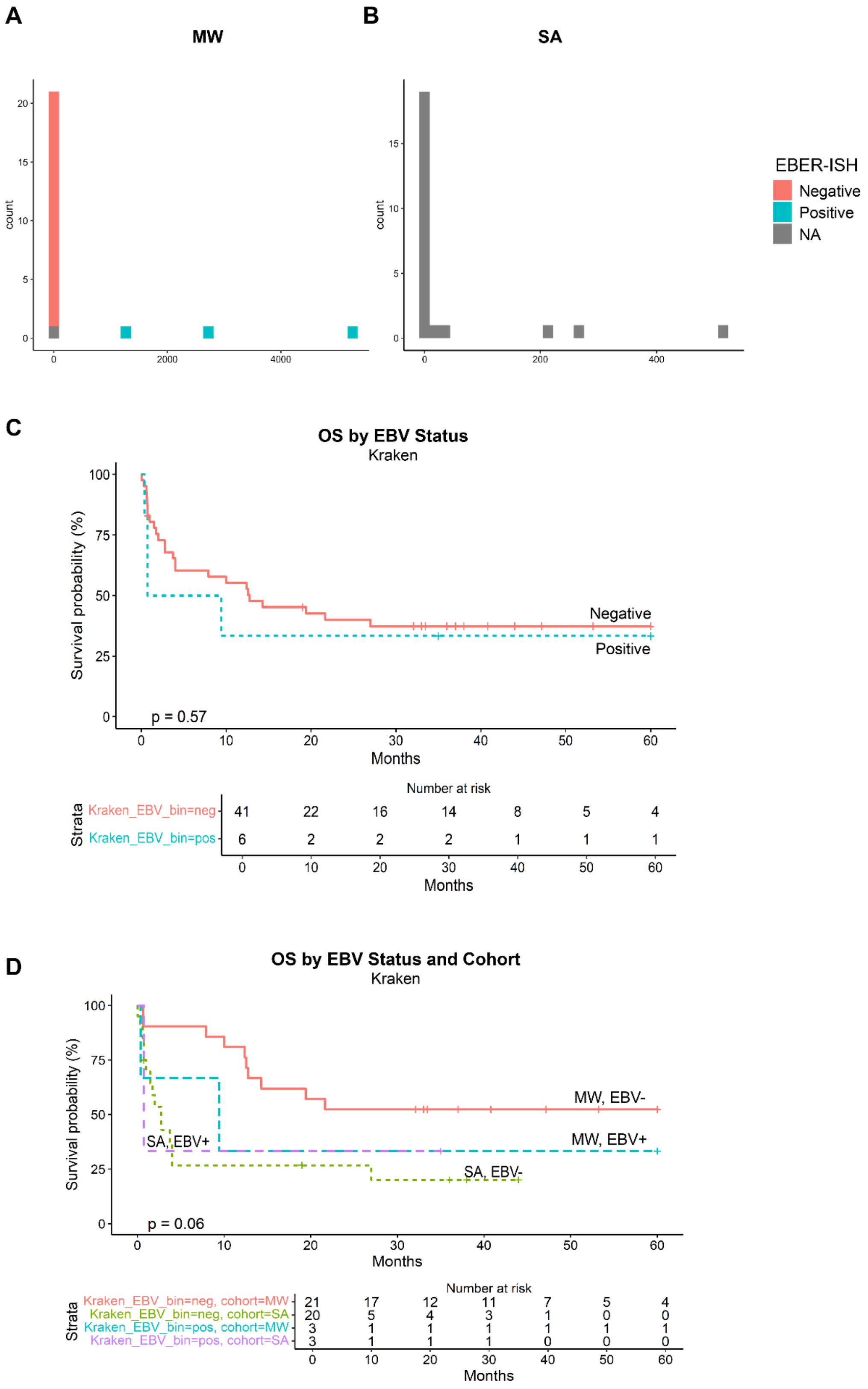

Supplemental Figure 4. EBER-ISH compared to EBV viral count and EBV viral count by overall survival. A) MW EBV viral count by Kraken, colored by EBER-ISH (purple = EBER-ISH positive). **B)** SA cohort EBV viral count by Kraken. **C)** OS by EBV viral count by Kraken >200. **D)** OS by EBV viral count by Kraken >200 and cohort.
